# Cumulative Error in Digital Workflows for Full-Arch Implant Rehabilitation: A Narrative Review

**DOI:** 10.3390/bioengineering13020219

**Published:** 2026-02-13

**Authors:** Hao-Ting Chen, Sheng-Wei Feng, Thi Thuy Tien Vo, Yung-Li Wang, Fang-Yu Fan, I-Ta Lee

**Affiliations:** 1 School of Dentistry, College of Oral Medicine, Taipei Medical University, Taipei 11031, Taiwan; b202108058@gmail.com (H.-T.C.); shengwei@tmu.edu.tw (S.-W.F.); cetuswang@tmu.edu.tw (Y.-L.W.); 2 Faculty of Dentistry, Nguyen Tat Thanh University, Ho Chi Minh City 700000, Vietnam; vtttien@ntt.edu.vn; 3 School of Dental Technology, College of Oral Medicine, Taipei Medical University, Taipei 11031, Taiwan

**Keywords:** computer-aided design, computer-aided manufacturing, dental implants, dental prosthesis, implant-supported, workflow

## Abstract

Despite the widespread adoption of digital technologies in modern implant dentistry, a comprehensive synthesis of error propagation across the entire workflow of full-arch implant rehabilitation remains absent. This narrative review aimed to synthesize current evidence on cumulative error propagation throughout the digital workflow of full-arch implant rehabilitation. Rather than focusing on isolated accuracy metrics, this article proposes a conceptual “Error Control Framework” to elucidate how minor deviations introduced at different workflow stages interact and amplify. A comprehensive literature search (2015–2025) was conducted to analyze error generation across five interrelated phases: Planning, Acquisition, Processing, Output, and Feedback. The evidence indicates that inaccuracies in full-arch implant rehabilitation behave as a cascading system (snowball effect) rather than isolated events. Errors introduced during early stages establish an irreversible baseline that is magnified during digital processing and manufacturing. Consequently, reactive verification at delivery alone is insufficient. To address this, this article proposes a proactive Error Control Framework that integrates a “Front-End Loading” strategy (necessitating strict upstream standardization of scanning strategies and scan-body geometry), alongside “Critical Control Points” (enforcing mandatory physical verification prior to final manufacturing). Viewing digital full-arch rehabilitation as a cumulative error system allows clinicians to implement preventive strategies and verification checkpoints, improving passive fit and long-term mechanical and biological outcomes.

## 1. Introduction

The rehabilitation of the completely edentulous jaw represents one of the most complex challenges in restorative dentistry. Historically, conventional analog protocols relying on physical impressions and stone casts have been the gold standard. However, these traditional workflows are often associated with patient discomfort, material distortion, and significant cumulative errors across the multiple laboratory stages involved [1].

In recent years, the continuous digitalization of surgical and restorative workflows has fundamentally reshaped this landscape. The integration of cone-beam computed tomography (CBCT), intraoral scanning, and computer-aided design/computer-aided manufacturing (CAD/CAM) has promised to enhance efficiency, standardization, and patient acceptance. These advances have enabled clinicians to virtually plan implant positions, design prostheses with high precision, and fabricate frameworks with minimal manual intervention. Recent consensus reports indicate that these digital advancements have successfully streamlined the workflow for single-tooth and partial rehabilitations, offering high predictability [1,2].

However, despite these technological leaps, the application of a fully digital workflow for full-arch implant rehabilitation remains a persistent challenge [1,2]. Unlike single-tooth cases, the edentulous arch lacks stable reference landmarks, making the digital acquisition and merging of datasets prone to deviation. Consequently, deviations within the digital workflow frequently compromise the passive fit of the final prosthesis [3]. Crucially, such discrepancies rarely stem from a single procedural failure but rather from the cumulative propagation of minor deviations throughout the workflow. This phenomenon creates a “snowball effect”: minor geometric inaccuracies during planning or intraoral scanning [4,5] can be amplified by registration mismatches in CAD processing [6], and further exacerbated by manufacturing tolerances such as toolpath offset or material shrinkage [7]. When these errors propagate through the chain, they accumulate beyond clinically acceptable limits, threatening the long-term success of full-arch implant rehabilitations [3,7].

From a clinical perspective, the “snowball effect” of error often manifests intraorally as framework non-passivity, generating residual static stress [3]. Clinically, this contributes to frequent technical complications, specifically screw loosening and chipping of the material [8,9,10]. While patient satisfaction often remains high despite these issues [8,9], the objective burden of care is significant. These complications necessitate unscheduled maintenance visits and increase long-term economic costs [10,11], thereby disrupting the patient’s daily life through repeated clinical interventions.

While extensive research has assessed the accuracy of isolated steps, including the trueness of intraoral scanners [12,13] and the fit of CAD/CAM frameworks, these data typically neglect the synergistic effect of errors across the entire workflow. Current literature offers limited insight into how upstream inaccuracies interact with downstream processing to impact the final clinical outcome. Although recent works have begun to quantify cumulative errors in specific in vitro scenarios [3,14,15], a comprehensive framework that integrates all workflow phases to guide clinical decision-making remains absent.

To bridge this knowledge gap, this narrative review synthesizes contemporary evidence on error propagation throughout the full-arch implant rehabilitation workflow. Unlike traditional reviews that segregate technologies, this article proposes an Error Control Framework structured around five interrelated phases: Planning, Acquisition, Processing, Output, and Feedback. This structure allows for the identification of critical error-prone stages and provides a unified strategy for clinicians to minimize cumulative discrepancies, ultimately enhancing the predictability of full-arch digital rehabilitations.

## 2. Methods

This narrative review was conducted following the recommendations of the Scale for the Assessment of Narrative Review Articles (SANRA) [16]. The aim was to synthesize evidence addressing accuracy, trueness, and error propagation within the digital workflow of full-arch implant rehabilitation, encompassing stages from planning to clinical feedback.

An electronic search was performed across PubMed (MEDLINE), Scopus, Web of Science, and the Cochrane Library databases. The primary search focused on studies published between January 2015 and October 2025 to ensure the analyzed data reflect the accuracy of contemporary digital hardware (e.g., current-generation intraoral scanners and CAM units) rather than obsolete technologies. To ensure comprehensive coverage and expand the research scope beyond electronic indexing, extensive manual searches of reference lists from key articles were also conducted to identify seminal works and relevant engineering studies. The search strategy utilized combinations of Medical Subject Headings (MeSH) and free-text terms. MeSH terms included: “Computer-Aided Design”, “Dental Implants”, “Dimensional Measurement Accuracy”, “Dental Prosthesis, Implant-Supported” and “Jaw, Edentulous”. These were combined with free-text keywords such as: (“full-arch” OR “complete-arch” OR “edentulous”) AND (“implant rehabilitation” OR “implant-supported” OR “dental implants”) AND (“digital workflow” OR “CAD/CAM” OR “intraoral scanning” OR “photogrammetry”) AND (“accuracy” OR “trueness” OR “precision” OR “error propagation” OR “passive fit”).

**Figure 1:**
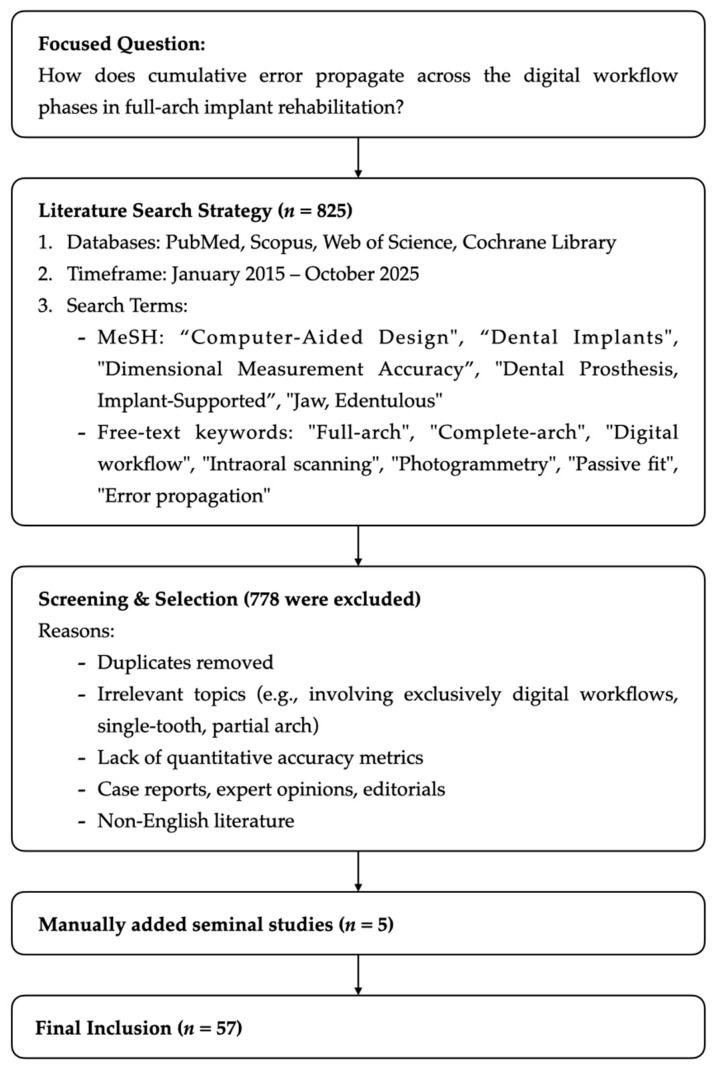
Flow chart describing the search process and selection of studies.

Initial screening was based on titles and abstracts, followed by a full-text evaluation. As detailed in Figure 1, a total of 825 articles were initially identified. After removing duplicates and applying exclusion criteria, 57 relevant sources were selected for the final synthesis. The selection process was conducted by two independent reviewers to minimize selection bias. Eligible articles included in vitro, in vivo, and clinical investigations, as well as meta-analyses that provided quantitative or qualitative evidence related to error generation or accumulation across workflow stages. Conversely, exclusions were applied to case reports, expert opinions, editorials, and studies involving exclusively digital workflows. Research limited to single-tooth or partial-arch restorations was also excluded, except in instances where it provided fundamental methodological insights critical to the validation of full-arch conventional protocols.

To ensure a structured analysis of the heterogeneous literature, data extraction focused on quantitative metrics of accuracy. For instance, root mean square (RMS) values for scanning deviations, linear and angular discrepancies for implant positioning, and marginal gap values for prosthetic fit were extracted and categorized according to the workflow phase (Planning, Acquisition, Processing, Output, Feedback). Given the narrative nature of this review, a qualitative synthesis approach was adopted. Rather than pooling statistical data, the synthesis focused on identifying causal linkages between upstream deviations and downstream clinical manifestations. Regarding quality assessment, a domain-specific criterion was applied: for in vitro studies, priority was given to investigations utilizing high-precision reference metrology (e.g., CMM); for clinical studies, preference was given to prospective cohorts reporting quantitative biological and mechanical complications.

## 3. Evidence Synthesis: Error Propagation Across Workflow Phases

Evidence from the reviewed literature indicates that small geometric or registration discrepancies introduced at one phase may influence subsequent steps, ultimately affecting the fit and biomechanical stability of full-arch restorations [3]. To clarify these interrelationships, the findings are presented according to a five-phase framework grounded in the data lifecycle of digital manufacturing (Table 1). This structure was specifically selected to trace the transformation of error as it moves from virtual definition (Planning) to digitization (Acquisition), computational manipulation (Processing), physical materialization (Output), and finally, clinical validation (Feedback). This approach allows for the isolation of specific error origins at each critical data transformation step.

### 3.1. Planning Phase

#### 3.1.1. Virtual Implant Planning

Virtual planning integrates anatomical CBCT data with prosthetic design [41]. At this stage, inaccuracies originate primarily from image resolution, segmentation thresholds, and registration between CBCT and surface scans [17,18,19,20]. Evidence from a study using Invivo 5 (Anatomage) for manual registration and OnDemand3D (Cybermed) and THEIA 1.0 (Genoray) for point-based registration demonstrated mean deviations of approximately 0.03–0.07 mm, with point-based methods outperforming manual alignment by nearly twofold [17]. However, metal artifacts can degrade this precision even with artifact-reduction tools (residual error ~0.32 mm) [18]. Regarding Field of View (FoV), while smaller volumes increase angular deviation, they remain clinically acceptable if sufficient teeth are retained to ensure tripodization [19].

The challenge of registration is amplified in fully edentulous arches due to the lack of dental landmarks. For dual-scan protocols, evidence indicates how different fiducial markers (e.g., gutta percha, metallic spheres) and guide-imaging methods (CBCT vs. intraoral scanner) impact planning-stage registration accuracy [20]. The traditional dual-CBCT-scan protocol (DICOM) approach yields consistent accuracy. Conversely, substituting the second CBCT with an intraoral scan of the guide significantly reduces accuracy, especially when gutta percha and resin were employed. Additionally, automatic alignment frequently fails, forcing a reliance on manual, operator-dependent alignment to complete the registration.

Artificial intelligence (AI) seeks to minimize operator variability. Recent studies report that AI-generated plans achieve clinical acceptability comparable to experts (approx. 89–93%) while reducing planning time by over 50% [41,42]. However, to the best of current knowledge, published evidence is largely limited to preclinical or single-tooth applications, necessitating further validation for full-arch workflows.

#### 3.1.2. Guided Implant Surgery Modalities

Regarding surgical execution, the reviewed literature consistently identifies Static Computer-Assisted Implant Surgery (s-CAIS) as the primary modality for transferring digital plans [21,22,43]. Within this domain, Raico Gallardo et al. highlighted that template stability differs significantly by support type [21]. Specifically, mucosa-supported guides, typically requiring multi-pin fixation, achieve significantly higher accuracy than bone-supported templates, which exhibit the greatest displacement [21]. Collectively, guide fit, tissue resilience, and sleeve tolerance can amplify upstream digital errors, particularly when extensive edentulous spans are involved [21,22,23,43].

To prevent these template-related limitations, dynamic navigation transfers the plan without a physical template and provides real-time feedback on the drill trajectory. In immediate full-arch loading, Pozzi et al. reported mean platform, apex, and angular deviations of 1.17 mm, 1.30 mm, and 2.19°, respectively [5]. Combining modalities enhances precision; Lorwicheanrung et al. reported that a synergistic approach (static guide + dynamic feedback) achieved a median angular error of approximately 0.60°, which was statistically superior to using either static CAIS (3.05°) or dynamic CAIS (3.24°) alone [23]. Robotic-assisted systems extend computer guidance by mechanically controlling the drill according to the digital plan. Wang et al. reported sub-millimeter deviations (0.65 mm/1.43°), outperforming static guidance [24]. Although promising, these findings derive from limited cohorts. Also, calibration, anchor-pin fixation and equipment cost remain important constraints.

While most high-precision data still originate from single-tooth or partial-arch models, the available full-arch evidence, including the prospective studies of Pozzi et al. and Lorwicheanrung et al., supports a substantial improvement in accuracy and reproducibility compared with conventional free-hand techniques [5,23].

### 3.2. Acquisition Phase

Regarding the acquisition phase, the reviewed literature consistently identifies the digitization process as a critical control point [26,44,45]. Specifically, studies categorize the factors influencing accuracy into patient-related anatomic limitations and operator- or device-dependent variables.

#### 3.2.1. Patient-Related Factors Influencing Digital Data Capture

Regarding patient-specific variables, evidence indicated that the absence of fixed reference landmarks and the presence of mobile soft tissue significantly predispose the dataset to geometric distortion [44]. In vitro investigations identify extended inter-implant spans and non-parallel implant angulations as critical geometric risk factors. Evidence consistently demonstrates that precision decreases and linear errors accumulate as the inter-implant distance increases, likely due to stitching inaccuracies over long spans. Furthermore, implant angulation significantly impacts trueness; non-parallel implants (up to 30°) have been shown to yield significantly lower accuracy compared to parallel configurations [25,26,27].

Challenge often lies in the lack of fixed references required for accurate image stitching. Accuracy is largely determined by the availability of stable anatomical features rather than jaw type alone. The mandible is frequently problematic due to environmental instability (movable mucosa, saliva) and a lack of landmarks, resulting in lower precision compared to the maxilla [46,47]. While the maxilla often benefits from palatal rugae that aid stitching, its wider arch span can conversely lead to higher accumulated linear deviations (exceeding 100 µm) in the absence of distinctive features [13].

Soft tissue thickness and surface humidity also impose critical constraints. Deeper implant placement (simulating a 4 mm soft tissue depth) reduces the visible geometry of the scan body, significantly impairing acquisition accuracy [28]. Additionally, wet environments consistently degrade data quality; simulated saliva has been shown to significantly increase angular discrepancies compared to dry conditions [27].

#### 3.2.2. Operator- and Device-Related Factors

While operator experience may be less critical with modern intraoral scanners compared to traditional methods [48], strict adherence to protocols remains significant. The choice of scanning strategy is a primary determinant; a study found a circumferential pattern superior to a zigzag pattern [29]. Regarding kinematics, while voluntary pauses were found to improve trueness by allowing data consolidation [30], artificial manipulations such as surface-locking patterns (freezing a previously scanned area) significantly reduced trueness, particularly when applied mid-arch (up to ~350 µm) [49]. Furthermore, excessive vertical rotation of the scanner head reduced trueness, resulting in mean deviations of approximately 100 µm, whereas a horizontal, continuous motion preserved it [31].

Studies indicate that scanner-specific reconstruction algorithms introduce intrinsic variability, with reported mean errors ranging broadly (16–120 µm) even under identical conditions [26,50]. More critically for full-arch rehabilitation, scanners exhibit heteroscedasticity, where the magnitude of error increases progressively with the length of the measured span [25].

To overcome the intrinsic limitations of stitching algorithms in long-span arches, recent protocols advocate for implementing an intervention during acquisition. Rustichini et al. demonstrated the efficacy of a “calibrated splinting framework”. By scanning a splint with known metrological coordinates, the acquisition data acts merely as a reference, while the definitive accuracy is derived from the pre-calibrated file [51], which allows the acquisition data to serve merely as a reference, deriving definitive accuracy from a pre-verified geometric file.

Alternatively, photogrammetry utilizes vector-based triangulation rather than image stitching to minimize progressive error accumulation [52]. Studies confirm photogrammetry achieves higher trueness and precision (often sub-70 µm) than both conventional impressions and intraoral scanning, regardless of span or splinting [45,47,53]. A in vitro comparison quantified this, finding photogrammetry systems (median deviations 25 µm) achieved the highest 3D trueness, followed by conventional splinted impressions, with the intraoral scanner demonstrating the lowest accuracy [54]. However, the inability to capture soft tissue necessitates integration with IOS datasets, adding workflow complexity and cost.

Regarding auxiliary components, Polyetheretherketone (PEEK) scan bodies consistently produce lower deviations than titanium due to favorable optical properties [55,56]. Geometric aids also enhance accuracy; prefabricated auxiliary devices connecting scan bodies provide stable references, significantly improving trueness and precision in edentulous scans [57]. Finally, the “segmented digital impression” (SgDI) technique involves capturing multiple, independent short-span scans, each recording an implant and a portion of a stable geometric pattern. These segments are then assembled in the CAD software using the pattern as a common reference, yielding significantly higher trueness and precision than a standard continuous scan [58].

### 3.3. Processing Phase

In the processing phase, the reviewed literature addresses error generation across three key digital domains: data integration for virtual patient creation, intrinsic algorithmic calculations within CAD environments, and predictive modeling during the design stage.

Regarding data integration, integrating facial scans to create a virtual patient introduces significant error primarily during the alignment process. Studies report substantial trueness variations in virtual patients, ranging from 0.50 mm to 1.64 mm depending on the alignment strategy and landmarks used [32,59]. Direct evidence of error amplification has been documented in vivo; Al Hamad et al. demonstrated that manual alignment increased the baseline facial scan error (MAD = 0.80 mm) to a final integrated error of 1.21 mm [33]. As for functional registration, digital bite accuracy frequently decreases toward posterior regions due to model tilting [60]. To mitigate this, a bilateral scanning protocol has been shown to reduce the tilting effect [61].

In addition to registration variables, errors at this stage also stem from the interaction between input data distortions and the proprietary algorithms of the CAD software. Pan et al. characterized the CAD process as a “closed black-box,” noting that internal calculations, particularly library matching or best-fit alignment, are automated [3]. Regarding specific error sources, Al-Meraikhi et al. identified the conversion of raw point clouds into continuous surfaces as a source of intrinsic approximation error [34]. Their findings indicate that when the software aligns a standard implant library component to a mesh, the algorithm generates coordinates based on mathematical best-fit rather than absolute anatomical truth, creating a potential positional misalignment during the design phase.

The digital design phase offers an opportunity for quality control. Tribst et al. utilized 3D-Finite Element Analysis (FEA) to quantify the impact of design parameters on mechanical stability. Their analysis revealed that specific modifications to the macro-level framework geometry resulted in a measurable reduction in stress concentration, decreasing values from 24.31 MPa in the conventional design to 13.27 MPa in the optimized model [62]. Furthermore, Fu et al. investigated the link between upstream data quality and downstream fit. Their study established a strong statistical correlation (R_2_ = 0.8–1.0) between the root mean square (RMS) values of the initial scan deviation and the marginal misfit of the final physical framework [15]. Based on this correlation, the study concluded that evaluating RMS values at the processing stage can serve as a reliable quantitative indicator of accuracy before manufacturing.

### 3.4. Output Phase

In the output phase, the reviewed literature focuses on two main areas: the comparative accuracy of different Computer-Aided Manufacturing (CAM) technologies and the methods for verifying the final prosthetic fit. Regarding the propagation of inaccuracy, Pan et al. indicated that the physical output represents the sum of errors accumulated from planning, acquisition, and processing, compounded by the specific deviations of the CAM process itself [3].

#### 3.4.1. Manufacturing Variables

Manufacturing accuracy varies considerably across technologies. In subtractive manufacturing (SM), both titanium and zirconia frameworks showed similar 3D distortion patterns, with mean deviations remaining under 90 µm [34]. Similarly, regarding additive manufacturing (AM), complete-arch titanium frameworks fabricated via selective laser melting (SLM) and electron beam melting (EBM) exhibit three-dimensional discrepancies ranging from 60 to 69 µm, with minimal z-axis distortion (6–11 µm) [35].

Despite these acceptable baselines for both technologies, subtractive methods generally outperform additive manufacturing for full-arch metal frameworks in direct comparisons. Studies confirmed that CNC-milled frameworks achieved significantly smaller marginal gaps than those produced by SLM, attributing the discrepancy to thermal stresses and layering roughness inherent in AM processes [63,64].

Interestingly, this superiority of milling is highly material-dependent. In contrast to metal workflows, AM resin frameworks exhibited superior marginal fit, particularly at distal abutments where SM frameworks showed a trend of increasing error [36]. However, this apparent advantage was counterbalanced by a process-specific limitation: AM frameworks demonstrated lower trueness on occlusal surfaces, an inaccuracy attributed to the support structures required during printing [36]. To mitigate these limitations, hybrid workflows combining additive fabrication with subsequent precision milling of the implant–framework interface have been reported to achieve accuracy comparable to, or even exceeding, conventional subtractive manufacturing [65].

#### 3.4.2. Clinical Manifestation, Verification and Error Interception

To verify the ultimate metric of clinical passivity, objective scientific methods have been developed to quantify the degree of fit beyond standard clinical assessments. Rutkunas et al. used a Screw Resistance Test to record the degrees of rotation required to torque the screw from initial seating (5 Ncm) to final tension (35 Ncm). While in vivo screw rotation angles appeared similar between groups (*p* = 0.557), master cast measurements showed that digital frameworks required significantly higher rotation to achieve closure than conventional ones (*p* < 0.05) [37], suggesting that the marginal integrity verified on the definitive cast may not be reliably predict true intraoral passivity.

Furthermore, Fouda et al. demonstrated that despite using a fully digital workflow, standard clinical tests flagged 60% to 80% of the frameworks as non-passive. This high failure rate was corroborated by objective micro-CT analysis, which confirmed that only 30% of the frameworks truly achieved an acceptable fit [14]. Consequently, the data demonstrated that a substantial proportion of misfits remained undetected when evaluated solely by conventional clinical criteria.

### 3.5. Feedback Phase

The Feedback phase represents the post-delivery period dedicated to long-term monitoring and maintenance. The reviewed literature focuses on clinical performance, mechanical integrity, and biological stability over observation periods ranging from 1 to 5 years [60,61,62,63]. In a randomized clinical trial comparing conventional and digital impression workflows for full-arch screw-retained maxillary rehabilitations, no significant differences were observed in prosthetic or biological outcomes [66]. However, despite the availability of such prospective evidence, the overall feedback frameworks described in the literature remain predominantly retrospective.

Regarding mechanical stability, the feedback phase serves to confirm the long-term durability of passive fit. Scarano et al. reported that 100% of CAD/CAM titanium bars maintained a passive fit over 5 years. However, latent errors manifested as complications: while survival was 100%, 3.33% of occlusal screws required re-tightening and 6.25% of cases required significant occlusal adjustment [38]. Corroborating these findings, Klein et al. reported that despite initial passivity, incidences of debonding and zirconia fracture were recorded, highlighting the risks in patients with heavy occlusal forces or parafunctional habits, underscoring the need for personalized occlusal monitoring [39]. To objectify such monitoring, Rodríguez Torres et al. validated a digital protocol utilizing serial intraoral scans. Although their study focused on partial edentulism, the methodology of aligning sequential STL datasets using reverse engineering software successfully quantified wear volume over time [67]. Applying this “serial superimposition” concept to full-arch rehabilitation represents a potential clinical protocol, while simultaneously offering a valuable research methodology to track material fatigue.

Long-term feedback also monitors biological stability, primarily through marginal bone level (MBL) changes. De Angelis et al. observed that MBL stabilized following an initial remodeling period (0.32 ± 0.05 mm at 5 years) [40]. Furthermore, Cappare et al. found no statistically significant difference in bone loss between digital (1.11 ± 0.54 mm) and conventional (1.07 ± 0.66 mm) workflows at the 2-year follow-up. Collectively, high implant and prosthetic survival rates (98–100%) were consistently reported [60,61,62,63].

Currently, while the application of digital monitoring protocols for full-arch cases remains under-reported, specific protocols have shown promise for retrospectively quantifying error. Jokstad & Shokati validated a digital diagnostic protocol by digitally aligning STL datasets obtained from intraoral scans of abutments and desktop scans of the prosthesis’s intaglio surface. Their analysis revealed that while misfit (up to approx. 230 µm) demonstrated a weak correlation with marginal bone loss, it was significantly associated with a history of mechanical complications, specifically screw loosening or fracture [68].

## 4. Discussion

### 4.1. Mechanisms of Error Propagation and Material Sensitivity

The cumulative error in full-arch rehabilitation acts as an amplifying system. Consequently, upon clinical delivery, forcing the physical framework into place converts this geometric mismatch into residual static stress [3].

This phenomenon of shifting error sources is particularly evident during the surgical phase. Comparatively, while free-hand placement consistently yields the largest deviations [69], digital guidance methods consistently reduce error to approximately 1 mm and 3° [5,23,24]. However, even within these digital modalities, the error pathways shift: static Computer-Assisted Implant Surgery (s-CAIS) typically suffers from template instability, whereas dynamic and robotic systems eliminate the guide but introduce new dependencies on registration and tracking calibration. The absence of a printed guide eliminates fabrication tolerance but introduces dependence on registration precision, optical tracking, and operator eye–hand coordination.

Within this cascading system, the Acquisition phase constitutes the most critical bottleneck. Unlike manufacturing errors, which are generally systematic and compensable, errors introduced during intraoral scanning, exemplified by deviations caused by mucosal mobility or stitching distortion over long spans, are irregular and difficult to correct downstream [25]. As a result, deviations at this stage establish a flawed foundation for the entire rehabilitation process. Although newer systems (e.g., Trios 5, Primescan 2) have introduced hardware refinements, current comparative evidence largely reflects earlier generations, suggesting that progressive error accumulation remains a persistent challenge.

Following acquisition, the evidence underscores that each registration step, encompassing both aesthetic facial scanning and functional bite registration, slightly displaces coordinates. This cumulatively distorts the digital foundation before the design phase even begins. Subsequently, the opacity of proprietary CAD algorithms obscures critical steps, such as the conversion of point clouds to surfaces. Consequently, input discrepancies are processed through these “black box” operations, generating secondary distortions that embed irreversible errors into the final prosthesis design. This is particularly evident in the creation of a virtual patient. Evidence indicates that the alignment process itself can significantly exacerbate baseline facial scan errors [33]. Crucially, this level of inaccuracy currently renders the virtual patient workflow unreliable for definitive manufacturing, restricting its application primarily to preliminary planning.

Furthermore, the clinical impact of this cumulative error is modulated by the prosthesis type. Rigid monolithic materials, such as zirconia, exhibit minimal elastic deformation and consequently possess a lower tolerance for cumulative error. In these scenarios, minor geometric misfits translate directly into destructive peak stresses, thereby increasing the risk of screw loosening or component complications [8]. Conversely, hybrid prostheses (e.g., metal-acrylic) offer a degree of resilience; the polymeric material can absorb a portion of the strain induced by framework misfit. While this may mask the underlying inaccuracy, it frequently manifests as chipping or wear over time [9,10].

### 4.2. Clinical Implementation: The Error Control Framework

**Figure 2:**
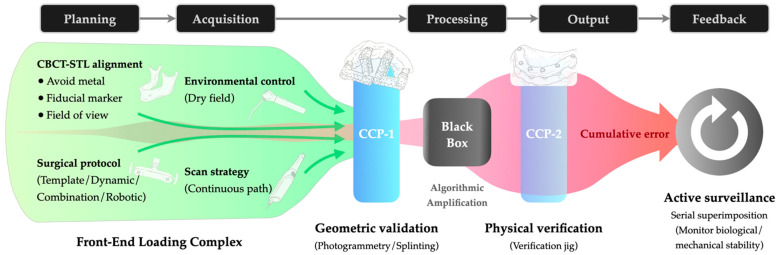
Proactive Error Control Framework in full-arch digital rehabilitation: The schematic illustrates the mitigation of cumulative error (Red zone) through Front-End Loading (Green zone; preventative protocols) and Critical Control Points (Blue bars; interception gates). CCP-1 performs geometric validation before data processing (“Black Box”), while CCP-2 performs physical verification before final output. (Abbreviations: CBCT, Cone-Beam Computed Tomography; STL, Standard Tessellation Language; CCP, Critical Control Points).

To mitigate the cumulative error effect, the traditional reactive approach of relying solely on visual checks at the final delivery stage has proven insufficient [14]. Moreover, in fully digital workflows, the absence of physical models prevents tactile verification, allowing errors to propagate undetected. Consequently, latent deviations often surface only at the final clinical try-in, making rectification costly. To address this, we propose a proactive Error Control Framework (Figure 2) grounded in two complementary strategic principles: Front-End Loading (FEL) and Critical Control Points (CCPs). FEL functions as a mechanism of “prevention” to minimize error generation at the source, while CCPs serve as a mechanism of “interception” to block error propagation before it becomes irreversible.

Since registration errors in the Planning phase and scanning distortions in the Acquisition phase are largely non-correctable downstream, FEL necessitates strict upstream standardization. Operationalizing this principle requires rigorous protocols, particularly regarding the verification of CBCT-STL alignment. For instance, retaining terminal dentition serves as a strategic advantage, balancing radiation dose reduction (via smaller Field of View) with the need for widely distributed fiducial markers [19]. Furthermore, standardization of the scanning protocol is paramount to minimizing the accumulation of stitching errors. This entails strict adherence to scanning parameters, such as utilizing continuous paths and humidity control. These measures minimize input error before it enters the amplification stages.

However, acknowledging that some degree of error propagation is inevitable in complex digital workflows, FEL alone is insufficient; the continuous chain must be physically interrupted for validation. This necessitates the implementation of Critical Control Points (CCPs), mandatory checkpoints where the digital workflow pauses to validate accuracy against physical reality. Conceptually, this approach establishes a “Predictive Diagnosis” mechanism. By evaluating dataset trueness before CAD execution (CCP-1), clinicians may predict and intercept unacceptable fabrication errors prior to manufacturing, rather than detecting them at delivery. Key strategies include using a calibrated splinting framework or photogrammetry during Acquisition to intercept stitching errors, and employing a verification jig during the Output phase prior to final finishing to validate the cumulative accuracy against the patient’s anatomy.

Finally, the framework closes the error control loop through Active Surveillance in the Feedback phase. By utilizing digital diagnostics such as serial scan superimposition, this stage objectively quantifies material fatigue and persistent errors. This shifts the clinical protocol from passive observation to active monitoring, ensuring long-term biological and mechanical stability.

### 4.3. Future Directions: From Qualitative Synthesis to Quantitative Decision-Making

While this narrative review qualitatively outlines the mechanisms of error propagation, the current literature exhibits significant heterogeneity in reporting metrics, preventing direct quantitative ranking of all available digital systems. To bridge this gap, future research must prioritize the standardization of data reporting (e.g., consistent use of RMS, angular deviations, and time-cost metrics). Establishing such homogenous datasets is a prerequisite for applying Multi-Criteria Decision-Making (MCDM) approaches, such as the R-method [70], which relies on structured experimental data. Integrating these quantitative ranking frameworks into the digital implant workflow could allow clinicians to objectively select scanning and manufacturing devices based on a weighted balance of precision, speed, and cost, thereby proactively controlling the error baseline.

### 4.4. Limitation

This narrative review acknowledges several limitations. First, the reviewed literature exhibits significant heterogeneity in study design; consequently, direct comparisons between in vitro findings and clinical outcomes must be interpreted with caution due to the confounding influence of in vivo environmental variables such as saliva and patient movement. Second, a scarcity of long-term randomized controlled trials specifically comparing the biological complications of fully digital versus conventional workflows for full-arch rehabilitation persists. Future research should prioritize longitudinal clinical studies to further validate the correlation between digital error accumulation and long-term prosthetic survival.

## 5. Conclusions

The central finding of this narrative review is that the final accuracy of a full-arch prosthesis is not determined by the precision of any single device, but by the integrity of the entire error propagation chain. Evidence confirms that deviations in full-arch rehabilitation act as a cascading system rather than isolated events. An initial, often imperceptible deviation in the Planning phase establishes an irreversible baseline, which is subsequently magnified by stitching algorithms during Acquisition and amplified by “black box” calculations during the Processing phase, before finally manifesting as physical misfit in the Output phase. Crucially, the Acquisition phase is identified as the primary source of irreversible error. Clinicians should prioritize accuracy at this stage and select restorative materials, whether rigid or resilient, based on the patient’s specific risk profile for complications.

Based on the synthesized evidence, a proactive Error Control Framework integrating Front-End Loading and Critical Control Points is proposed to mitigate the irreversible snowball effect. Ultimately, achieving predictable passive fit in full-arch rehabilitation requires a paradigm shift. Clinicians must move away from a reliance on the advertised precision of individual hardware and adopt a systematic verification protocol. By transforming the digital workflow from an unpredictable process into a series of verifiable checkpoints, the cumulative error can be effectively managed, ensuring long-term biological and mechanical success.

## Figures and Tables

**Table 1:** Summary of key error sources, contributing factors, and associated references across the five phases of the digital workflow of full-arch implant rehabilitation.

Phase	Specific Error Source	Key Contributing Factors/Mechanism	Reference
Planning	Image Registration	CBCT-to-Surface scan alignment algorithm (Point-based vs. Manual)	Park et al. [17]
	CBCT Quality	Metal artifacts affect DICOM-STL registration	Alhossaini et al. [18]
	Field of View (FoV)	Small FoV limits triangulation points, reducing precision	Hamilton et al. [19]
	Fiducial Markers	Marker material (Radiopaque vs. Metallic) and Guide scanning method (CBCT vs. IOS)	Ressurreição et al. [20]
	Surgical Template	Mucosa resilience (instability), Sleeve tolerance, Fixation pin movement	Raico Gallardo et al. [21] Kasradze et al. [22] Lorwicheanrung et al. [23]
	Dynamic/Robotic	Tracking calibration, Registration shifts, Operator hand-eye coordination	Pozzi et al. [5] Wang et al. [24]
Acquisition	Scan Stitching	Cumulative error over long inter-implant spans	Zingari et al. [25] Mangano et al. [26]
	Jaw Morphology	Lack of landmarks	Kernen-Gintaute et al. [13]
	Implant Geometry	Non-parallel angulation and Deep placement depth	Gómez-Polo et al. [27] Tawfik et al. [28]
	Scan Strategy	Path selection, Surface humidity, Kinematics	Gómez-Polo et al. [29] Limones et al. [30] Oh et al. [31]
Processing	Data Integration	Facial scan alignment and Virtual patient accuracy	Revilla-León et al. [32] Al Hamad et al. [33]
	CAD Algorithms	Library coordinate mismatch, Meshing distortion	Pan et al. [3] Al-Meraikhi et al. [34]
Output	Milling (SM)	Toolpath offset, Machine calibration	Al-Meraikhi et al. [34]
	Printing (AM)	Thermal stress (Metal), Support artifacts (Resin)	Revilla-León et al. [35] Yilmaz et al. [36]
	Verification	Subjectivity of visual/tactile checks (undetected passive misfit)	Rutkunas et al. [37] Fouda et al. [14]
Feedback	Latent Complications	Screw loosening, Material fatigue, Biological complications	Scarano et al. [38] Klein et al. [39] De Angelis et al. [40]

## Data Availability

No new data were created or analyzed in this study.
